# Coagulase-Negative Staphylococci Determined as Blood Culture Contamination Have High Virulence Characteristic Including Transfer of Antibiotic Resistance Determinants to *Staphylococcus aureus* and *Escherichia coli*

**DOI:** 10.3390/ijms26094424

**Published:** 2025-05-06

**Authors:** Bartosz Rybak, Olesia Werbowy, Karol Debowski, Magdalena Plotka, Aleksandra Maria Kocot

**Affiliations:** 1 Department of Environmental Toxicology, Faculty of Health Sciences with Institute of Maritime and Tropical Medicine, Medical University of Gdansk, Debowa 23A, 80-204 Gdansk, Poland; bartosz.rybak@gumed.edu.pl (B.R.); k.debowski@gumed.edu.pl (K.D.); 2 Department of Microbiology, Faculty of Biology, University of Gdansk, Wita Stwosza 59, 80-308 Gdansk, Poland; olesia.werbowy@ug.edu.pl

**Keywords:** coagulase-negative staphylococci (CoNS), antibiotic resistance, skin disinfection, blood culture contamination BCC, conjugation, virulence

## Abstract

This study aimed to evaluate the virulence of 36 clinical isolates estimated as blood culture contaminants (BCCs). MALDI-TOF MS classified all isolates as coagulase-negative staphylococci (CoNS) with the highest percentage of S. epidermidis (77.78%). All tested strains formed biofilms with greater ability at room temperature than 37 °C. CoNS were sensitive to vancomycin (0% resistance) and had relatively low resistance to linezolid and rifampicin (8.33 and 22.22% resistance). The highest resistance was observed for penicillin (94.44%). Moreover, we observed the transfer of antibiotic resistance genes from the tested CoNS to *S. aureus* and even to *E. coli*, although with lower efficiency. CoNS in planktonic form were completely combated by antiseptics after 10 and 60 s exposition, and activity against biofilms was time-dependent. The complete elimination of biofilms was observed after a 180 s exposure to Kodan and CITROclorex, and this exposure to Rivanol and Octenidyne showed still viable cells (>0.9 log CFU/mL). Our findings showed that a careful selection of antiseptics and extending the exposure time before blood collection can reduce the occurrence of blood culture contamination. However, our most important finding is the indication that CoNS naturally occurring on human skin and mucous membranes exhibit antibiotic resistance, and what is more, determinants of antibiotic resistance are transferred to both closely related Gram-positive bacteria and phylogenetically distant Gram-negative bacteria. Thus, our findings shed new light on CoNS—they indicate the necessity of their control due to the effective transfer of mobile genetic elements harboring antibiotic resistance genes, which may contribute to the spread of resistance genes and deepening the antibiotic crisis.

## 1. Introduction

Blood collection is a routine procedure practiced in hospitals and medical facilities, and blood cultures are the basic test for the diagnosis of sepsis, bacteremia and fungemia [1,2]. Bacteremia and fungemia are bloodstream infections (BSIs) that threaten human life and take a deadly toll every year around the world [3]. Bacterial BSI has a mortality rate over 20% and this rate can be minimized by the early and accurate diagnosis of BSI [4]. Even though blood culture is not a quick diagnostic method, it still remains the “gold standard” in BSI detection [4]. However, time consumption is not the only limitation of using blood cultures to detect bacteremia or sepsis. Despite blood collection in medical facilities by qualified medical personnel, the contamination of blood cultures is relatively common [5].

Blood culture contamination (BCC) is also referred to as a false-positive blood culture result and is defined as the presence of at least one set of bacteria commonly found on the skin, e.g., *Staphylococcus* sp., *Micrococcus* sp., *Aeromonas* sp., specified in the Cumulative Techniques and Procedures in Clinical Microbiology (CUMITECH) guidelines [6]. In other words, BCC is the phenomenon of bacteria presence in a blood sample that are not actually present in the patients’ bloodstream [6]. Monitoring the BCC rate is an indicator of the quality of the laboratory and medical staff collecting blood, and the Clinical and Laboratory Standards Institute (CLSI) recommends 3% as an acceptable rate of BCC [2]. A false-positive blood culture results in the unjustified use of antibiotics, which contributes to the increase in antibiotic resistance and forces the longer hospitalization of the patient, and this in turn causes economic losses [7]. The causes of BCC include the incorrect collection of blood samples, a lack of experience of the staff performing this procedure, failure to comply with aseptic rules, crowds at the place of blood collection, haste or the improper transport of samples [5].

Among the microorganisms reported as BCC, the most frequently isolated are coagulase-negative staphylococci (CoNS) with the highest percentage of *Staphylococcus epidermidis* [2]. CoNS naturally inhabit human skin and mucous membrane and their presence in blood cultures is most often the result of improper skin disinfection before blood collection [1]. They are opportunistic pathogens particularly virulent in immunocompromised patients, and in medical environments, CoNS cause nosocomial infections or BSI [8]. Nosocomial infections are observed in patients with indwelling medical devices such as catheters and prosthetic implants, and their consequence may be BSI [2,8]. Although the presence of CoNS in blood cultures is still most often the result of contamination, the number of cases of BSI caused by this group of bacteria is increasing [8]. For this reason, it is extremely important to distinguish BSI from contamination [9]. Over the years, various strategies have been attempted to verify blood culture results and classify the results as contamination or infection; however, there are still no precise tools to clearly exclude BCC and we still take actions related to an incorrectly detected BSI [10]. BCC is a risk not only in the context of the unjustified use of antibiotics. The increasing number of BSIs caused by CoNS indicates the virulence of these strains. There is no doubt about the virulence of the strains causing BSI, but the virulence factors seem to be unrelated to the strains identified as BCC, which naturally inhabit human tissues. In this context, one of the important aspects is the possibility of transferring resistance genes, and currently, the greatest challenge in medicine is antibiotic resistance [11]. Antibiotic resistance genes are located on mobile genetic structures such as plasmids, transposons, integrons and bacteriophages. Consequently, the potential antibiotic resistance of CoNS poses a risk of increasing the mobility of antibiotic resistance determinants, e.g., by horizontal gene transfer [12]. The easiest and most effective way to transfer antibiotic resistance between bacteria is conjugation, i.e., the direct transfer of resistance plasmids between bacterial cells [13]. Importantly, conjugation occurs in natural environments—bacteria can exchange plasmids, among other entities, in the intestines, sewage or hospitals, which contributes to the rapid spread of resistance [13]. The efficiency of conjugation may vary depending on whether it occurs between bacteria of the same species (homologous conjugation) or between unrelated bacteria (heterologous conjugation). Conjugation between unrelated bacteria is less efficient but not impossible [14]. However, research on the spread of resistance between Gram-positive and Gram-negative bacteria is limited. Therefore, although the problem of BCC is raised in the scientific literature, it most often concerns antibiotic resistance or biofilm formation by detected BCC. In this study, we undertook a multi-aspect assessment of CoNS identified as BCC. We assessed the ability of CoNS to form biofilms at different temperatures, examined their resistance to antibiotics and sensitivity to antiseptics considering planktonic cells and biofilms, and additionally assessed the possibility of transferring antibiotic resistance determinants to representatives of Gram-positive bacteria—closely related *Staphylococcus aureus* and to Gram-negative bacteria represented by *Escherichia coli*. The demonstration of effective conjugation between CoNS identified as BCC and other bacteria, especially the transfer of resistance determinants to phylogenetically distant *E. coli*, is a pioneering effect that has not been well described in the literature so far.

## 2. Results and Discussion

### 2.1. Identification of Blood Culture Contaminants

A total of 36 bacterial isolates were obtained as BCC with 17 isolates from UCC and 19 from CED patients (Table 1). The number of isolates in both groups was similar with a slight predominance in CED samples, where patients stayed for a maximum of 48 h. This result is consistent with previous literature reports, which indicate that the number of BCC cases is higher among samples from emergency departments. For instance, in the study by Robertson et al. [15], they reported that BCC in emergency departments accounted for 11.7%, while in other hospital departments, it was around 2.5% [15]. The higher percentage of BCC in blood samples from emergency departments may be due to the fact that these patients require quick, urgent and emergency help, often provided in chaotic and confusing circumstances and under time pressure [1].

The identification of isolates using MALDI-TOF MS classified all strains as *Staphylococcus* spp. (Table 2). The highest percentage of isolates (77.78%) was estimated by *S. epidermidis*, and this result was observed in both groups, where this strain accounted for 76.47% and 78.95% in the UCC and CED groups, respectively (Table 3). In second place was *S. haemolyticus* with a value of 8.33% among all isolates, while representatives of other species—*S. saprophiticus*, *S. hominis*, *S. capitis* and *S. simulans*—occurred once or twice in one or both groups (Table 3). It should be emphasized that all strains determined as BCC were coagulase-negative staphylococci (CoNS). These observations correspond to the previous literature data, where the most frequently observed microorganisms in BCC were CoNS. Additionally, representatives of *Micrococcus* sp., *Propionibacterium* sp., *Bacillus* sp. and *Corynebacterium* sp. are often reported as contamination [1]. Similar results were obtained in the study by Aiesh et al. [2], where staphylococci accounted for over 89% of all BCC and *S. epidermidis* constituted the largest percentage (49.2%) obtained in a retrospective three-year study performed at An-Najah National University Hospital (NNUH) [2].

In our study, we identified only CoNS, which is not surprising because staphylococci are saprophytic bacteria that inhabit the human skin and mucous membranes [2,16]. However, despite this, these opportunistic pathogens are one of the main causes of nosocomial infection (NI) and BSI [15,17]. In a hospital environment, various medical equipment, such as catheters, prostheses and valves, violate the continuity of human tissues and natural protective barriers and may cause extensive NI [8]. In recent years, the number of BSIs caused by the CoNS has been increasing, but this group of bacteria still remains the main cause of BCC. Therefore, it is important to distinguish true-positive results indicating infection from BCC to avoid the use of antibiotics without real reason to not extend hospitalization and to limit economic losses [10]. BCC seems to be seemingly harmless, but it should be remembered that hospitals often house immunocompromised patients for whom exposure to CoNS may pose a real threat. Therefore, in the next step, we decided to investigate the virulence of CoNS identified as BCC by assessing their ability to form biofilms, determining their sensitivity to conventional antibiotics, assessing the transfer of antibiotic resistance determinants to *S. aureus* and *E. coli* and evaluating their sensitivity to antiseptics used to disinfect the skin before blood collection.

### 2.2. Biofilm Formation by CoNS Determined as Blood Culture Contamination

**Figure 1:**
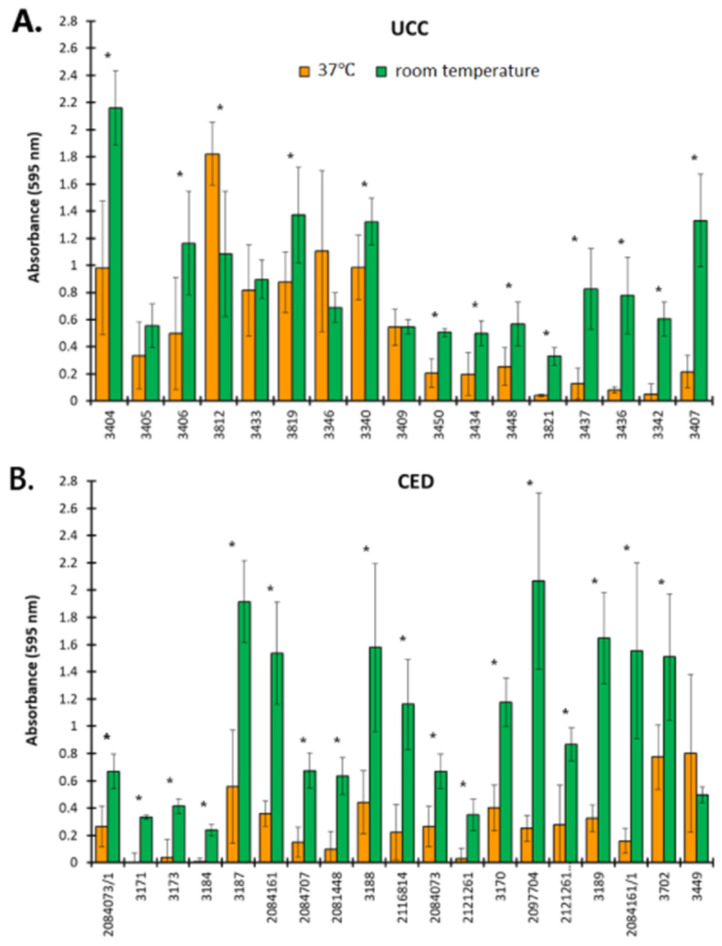
The ability to form biofilm by coagulase-negative staphylococci (CoNS). Strains isolated as blood culture contamination (BCC) from a patient of the University Clinical Center (UCC, (**A**)) and the Clinical Emergency Department (CED, (**B**)) in Gdansk. The results are obtained with crystal violet staining. Asterisks indicate statistically significant differences between the results obtained for strains at 37 °C and room temperature.

The ability of CoNS to form biofilms was evaluated according to Al-Bayati and Samarasinghe [18] and the results are presented in Figure 1. Isolated strains from both UCC and CED groups produced biofilm better at room temperature than at 37 °C. At room temperature, 15 strains from the UCC group (88.24%) were classified as strong biofilm producers and the remaining 2 strains (11.76%) as moderate biofilm producers (Figure 1A), while in the CED group, 14 strains (73.69%) were classified as strong biofilm formers, 4 (21.05%) as moderate and 1 (5.26%) as a weak biofilm producer (Figure 1B). For comparison, at a temperature of 37 °C in the UCC group, only seven strains (41.11%) showed strong biofilm-producing abilities and two (11.76%) showed moderate biofilm-forming properties (Figure 1A), and in the CED group, only three strains (15.79%) showed strong biofilm-forming abilities and four (21.05%) showed moderate biofilm-forming abilities (Figure 1B).

Previous reports indicate that CoNS clinical isolates are good biofilm producers. For instance, in the study by Shrehsta et al. [19], 51 (71.83%) out of all 71 CoNS showed the ability to form biofilms. Additionally, in the group of 71 isolates, *S. epidermidis* was the most frequently reported (40%, 28/71), and of *S. epidermidis*, 82% of isolates (23/28) had the ability to form biofilms. Among all 71 isolates, 17 came from blood, and among this group, biofilm-forming strains accounted for 70% (12/17) [19]. These results indicate high potential for biofilm formation in the hospital environment, which is also confirmed by the numerous recorded nosocomial infections caused by the presence and colonization of various surfaces, including medical devices, by CoNS [9]. Biofilm formation is one of the virulence factors. Bacteria in the biofilm are more resistant to antibiotics than planktonic cells, which may be dictated by the presence of an extracellular matrix, a higher density of bacteria, their slower growth, the difficult penetration of antibiotics into the deeper layers of the biofilm and/or the binding of antibiotics to biofilm components [15,16,19]. Thus, fighting biofilms is extremely difficult.

Our observations indicates that CoNS form more robust biofilms at room temperature than at 37 °C (*p* < 0.05 for most strains), although the optimum temperature for the growth of staphylococci oscillates in the range of 30–37 °C. However, these bacteria have the ability to grow at temperatures of 6.5–45 [20]. Moreover, there are various methods to determine the biofilm-forming abilities of bacterial strains [9,18,21]. For instance, in the study by Wojtyczka et al. [21], differences in the ability to form biofilms were observed depending on the method used. An assessment of the biofilm-forming properties of 122 strains isolated from the hospital environment revealed 9 strains (7.4%) forming biofilm based on the result of a Congo red assay, while a microtiter plate assay reported 12 (9.8%) strains with strong biofilm-forming abilities and 13 (10.7%) strains with an intermediate ability to biofilm formation [21]. In turn, in the study by Papadimitriou-Olivgeri et al. [9], among 57 strains of *S. epidermidis* identified as contamination or infection with the use of the microtiter plate method and on a glass surface, 37 (64.91%) and 24 (42.11%) strains showed the ability to form biofilms, respectively [9]. Moreover, regardless of the methods proposed by various authors to test the ability of CoNS to form biofilms, the temperature conditions ranged from 35 to 37 °C. Therefore, our research sheds new light on the problem of the formation of biofilms by CoNS, showing that they are a threat not only due to the adhesion and colonization of medical devices violating the continuity of human tissues but also other medical equipment and various surfaces in the hospital environment, such as doors, soap dishes, towel holders, faucet spouts, cabinet drawers, light switches, bath sink drains, etc., where they constitute a bacterial reservoir and pose a threat to patients [22,23,24,25]. Moreover, the colonization of such surfaces protects bacteria both from the host’s immune response and from antibiotic treatment. Thus, the fight against CoNS biofilms in such diverse places in hospitals is almost impossible. To sum up, our results point to two important aspects. On the one hand, they indicate that CoNS can form biofilms in various places in the hospital environment where antibacterial substances have difficult access and biofilms maintain a bacterial reservoir. On the other hand, it is worth noting that in our study at a temperature of 37 °C, the strongest ability to form biofilms was demonstrated mainly by *S. epidermidis* (five out of seven strong biofilm producers in the UCC group and all three strong biofilm producers in the CED group). *S. epidermidis* is the main colonizer of the surface of human skin, the temperature of which ranges from 33.5 to 36.9 °C [26]. In healthy people, *S. epidermidis* exists in the outer layers of the epidermis as a biofilm between keratinocytes [27]. Considering the above, the following question arises: can the careful preparation of the skin surface before blood collection reduce the number of skin bacteria responsible for BCC? Therefore, in the next step, we assessed the effectiveness of antiseptics used for skin disinfection against planktonic cells and biofilms formed by isolated CoNS.

### 2.3. Susceptibility of CoNS to Antibiotics

**Figure 2:**
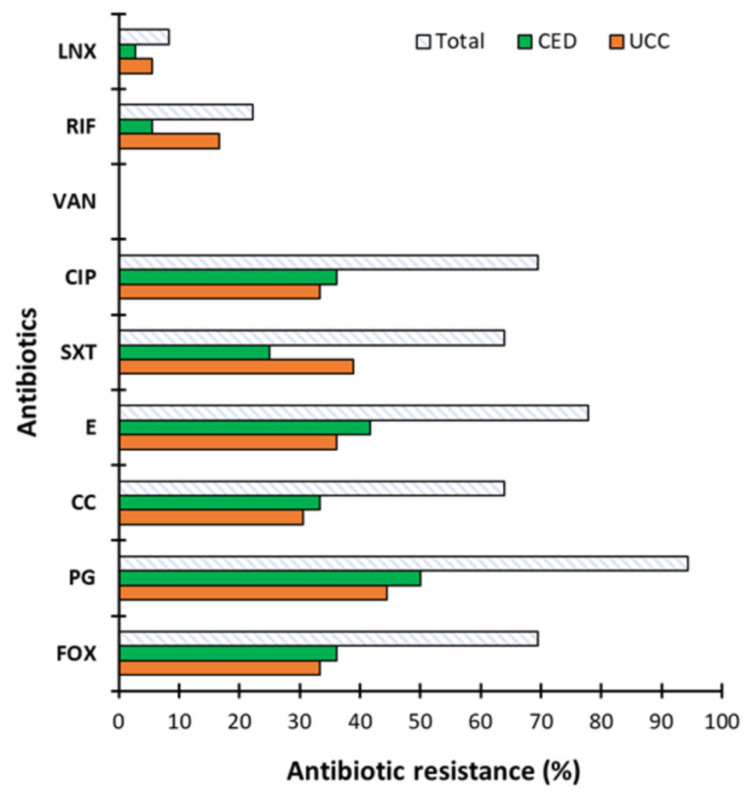
The antibiotic resistance of coagulase-negative staphylococci (CoNS). Strains determined as blood culture contamination (BCC) in patients of the University Clinical Center (UCC) and the Clinical Emergency Department (CED) in Gdansk. The antibiotics used are as follows: FOX—cefoxitin, PG—penicillin, CC—clindamycin, E—erythromycin, SXT—co-trimoxazole, CIP—ciprofloxacin, VA—vancomycin, Rif—rifampicin and LNX—linezolid. Results obtained using the disk diffusion method developed in accordance with Clinical Laboratory Standards Institute (CLSI) guidelines.

The sensitivity of CoNS from BCC to selected antibiotics is presented in Figure 2. It was shown that isolates from both groups were resistant to all tested antibiotics except VA (0% of resistant strains). CoNS were most resistant to PG with 94.44% resistant strains and least resistant to LNX with 8.33% resistant strains (Figure 2). For most of the antibiotics tested (CIP, SXT, E, CC, PG and FOX), resistant strains accounted for over 60% of all isolates. A lower percentage of resistant strains was observed for LNX and RIF (8.33 and 22.22%, respectively) and, importantly, CoNS from CED group were more sensitive to these two antibiotics than those from the UCC group (2.78 and 5.56% of strains resistant to LNX and RIF and 5.56% and 16.67% in the CED and UCC groups, respectively). Additionally, strains from the CED group were more resistant to five of nine antibiotics tested compared to the UCC group (CIP, E, CC, PG and FOX) with resistance in the range of 33.33–50.00%, while for the UCC group, their resistance was in the range of 30.56–44.44%.

**Figure 3:**
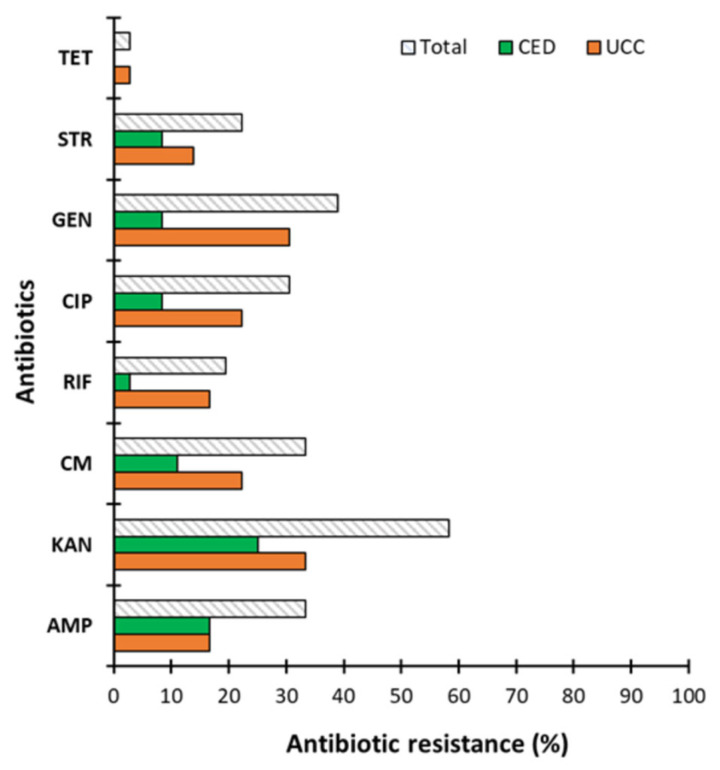
The antibiotic resistance of coagulase-negative staphylococci (CoNS). Strains determined as blood culture contamination (BCC) in patients of the University Clinical Center (UCC) and the Clinical Emergency Department (CED) in Gdansk assessed for antibiotics used at standardized microbiological concentration, where AMP—ampicillin, KAN—kanamycin, CM—chloramphenicol, RIF—rifampicin, CIP—ciprofloxacin, GEN—gentamicin, STR—streptomycin, and TET—tetracycline.

Similar trends were obtained for the standard microbiological concentration of antibiotics (Figure 3). The highest percentage of strains showed resistance to KAN (58.33%) with resistances of 33.33 and 25% for the UCC and CED groups, respectively. In turn, the lowest resistance was observed to TET (2.78%) and only in the UCC group.

The study of CoNS resistance to antibiotics has been carried out previously and the results also report the high antibiotic resistance of clinical isolates [9,28,29,30,31]. Our results correspond to previous scientific reports, which showed that CoNS are highly resistant to penicillin (>90%) but are sensitive to VA (0% antibiotic resistance strains) [32]. For instance, Papadimitriou-Olivgeri et al. [9] assessed the resistance of CoNS to 13 antibiotics, differentiating staphylococci into *S. epidermidis* and non-*S. epidermidis* isolated from blood. They showed that *S. epidermidis* were more resistant to most of the antibiotics tested. The authors observed no resistance to VA and DAP (daptomycin), while the highest percentage of resistant isolates was observed against FOX (cefoxitin), KAN (kanamycin) and FA (fusidic acid). Importantly, most strains showed multi-drug resistance [9]. It is worth emphasizing that both studies by Papadimitriou-Olivgeri et al. [9] and ours showed relatively low resistance to RIF, which is important due to the frequently reported synergy between RIF with VA and DAP, especially in the treatment of infections related to medical devices such as catheters [10,32]. The high percentage of antibiotic-resistant strains observed in previous studies and in our study poses a problem in combating staphylococcal infections. Additionally, many CoNS are multi-drug resistant, which increases the risk of the failure of antibiotic therapies [31]. The scientific data report no or a low resistance of CoNS to VA [31], which is consistent with our observations. For this reason, this antibiotic is often the drug of choice for serious infections, especially those associated with indwelling medical devices [33]. Nevertheless, vancomycin-resistant *S. epidermidis* (VRSE) is observed [34]. Considering the high antibiotic resistance of CoNS isolated as BCC, we decided to check the mobility of antibiotic resistance genes to other bacteria.

### 2.4. Mobilization Potential of Antibiotic Resistance Determinants

In order to assess the potential of the spread of antibiotic resistance genes from CoNS to other groups of bacteria, we decided to assess the mobility of antibiotic resistance determinants between different bacteria.

Plasmids have the largest impact in horizontal gene transfer. Among plasmid vectors, we can distinguish plasmids capable of autonomous transfer (conjugative) and requiring relaxation *in trans* (mobilizable). Conjugative plasmids can be narrow or broad host range. Typical narrow-host-range plasmids include, e.g., plasmid F (Inc F1) or R64 (Inc I1), which effectively mobilize mainly within *Enterobacteriaceae* [35]. In turn, among the broad-host-range plasmids, it is worth mentioning plasmid pIP501 (Inc18), which is involved in the propagation of vancomycin resistance from *Enterococci* to methicillin-resistant strains of *Staphylococcus aureus*. An interesting example is also a group of IncP-1α identical plasmids, RP4, RP1, RK2 and R68, which were detected, for the first time, in environmental isolates from multidrug-resistant bacterial isolates 56 years ago and are widely found in different hosts even now [36,37]. However, most bacterial plasmids use the mobilization *in trans* phenomenon, i.e., they do not encode complete conjugation machinery. They are mainly divided into plasmids encoding their own relaxation system coding by *mob* genes, such as ColE1 [37], or encoding only fragments of DNA sequences, mimicking *oriT* recognized by the relaxation systems of co-resident conjugative or mobilizable plasmids, e.g., pEC156 [38]. Including plasmids, which are extrachromosomal, autonomously replicating agents, an important aspect that improves the spread of antibacterial resistance determinants are integrative conjugative elements (ICEs). Similarly to conjugal plasmids, ICEs, for efficient DNA translocation, require most elements involved in conjugal transfer, plus a few additional enzymes, like transposases, integrases, which facilitate the unwinding and integration of DNA into a new host genome. SXT/R391 is the largest family of ICEs, detected in clinical and environmental isolates [39,40]. These mechanisms allow resistance genes to be inserted at multiple sites across diverse bacterial hosts, further amplifying the spread of resistance.

In this study, we evaluate the mobility of antibiotic resistance determinants between CoNS and other Gram-positive bacteria, particularly closely related strains, i.e., *S*. *aureus* (Table 4), and between a phylogenetically distant strain representing Gram-negative bacteria, i.e., *E. coli* (Table 5). Conjugation mechanisms in CoNS involve the relaxase-mediated processing of plasmid DNA, the assembly of Type IV secretion systems, and rolling circle replication, analogous to well-studied Gram-negative systems [41].

The transfer of resistance from CoNS to *S. aureus* occurred with high efficiency, and in most cases, the levels of resistance across different strains were similar (within a range of ±10), indicating that two or even three resistance features are transferred probably by one plasmid vector (Table 4). For *S. epidermidis* strains identified as 3437, 3212, 3214 and 3702 (CoNS), we observed that, alongside a likely self-transmissible plasmid (conjugative plasmid), mobilization transfer also occurs (Table 4). This was indicated by the similar high efficiency of two resistance features, whereas another vector demonstrated significantly lower efficiency, suggesting that it was a different plasmid vector. For the four mentioned strains, the efficiency dropped by at least 100-fold, indicating the involvement of a different plasmid vector (Table 4).

As expected, in *E. coli*, which is phylogenetically distant to CoNS, a transfer occurred in fewer cases (Table 5), yet the efficiency remained almost unchanged. This suggests that, in these cases, conjugative plasmids with a broad host range may be involved [42,43]. Broad-host-range conjugative plasmids facilitate resistance gene dissemination within and beyond CoNS species. Although precise molecular details in CoNS are still being elucidated, the presence of *tra* gene clusters and conjugative pili underscores the importance of conjugation in their resistance gene transfer [41].

The same resistance features were transferred in the same configurations, as seen in strains 3450, 3216, 2121261 and 3702. However, the plasmid from strain 3437 did not transfer, likely due to the incompatibility barrier of the conjugative or replication machinery (Table 5). Interestingly, strain 3216 transferred a combination of three resistance features, both to *S. aureus* and *E. coli* (Table 4 and Table 5).

**Figure 4:**
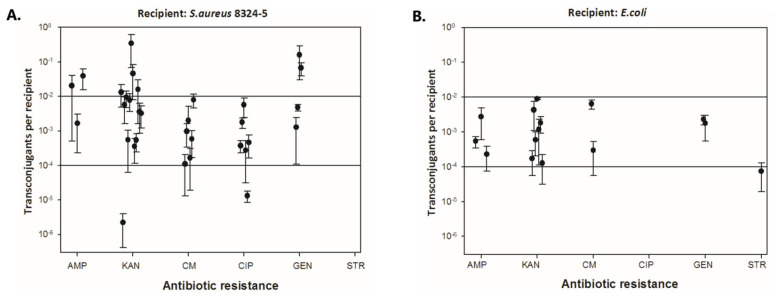
Efficiency of resistance gene transfer to individual antibiotics from coagulase-negative staphylococci (CoNS) to *S. aureus* 8324−5 (**A**) and *E. coli* (**B**).

Moreover, for the conjugation matings of CoNS-*S. aureus*, the transfer of determinants carrying resistance to all tested antibiotics was demonstrated (Figure 4A), while for the conjugation matings of CoNS-*E. coli*, no transfer of determinants encoding resistance to CIP was observed (Figure 4B).

**Figure 5:**
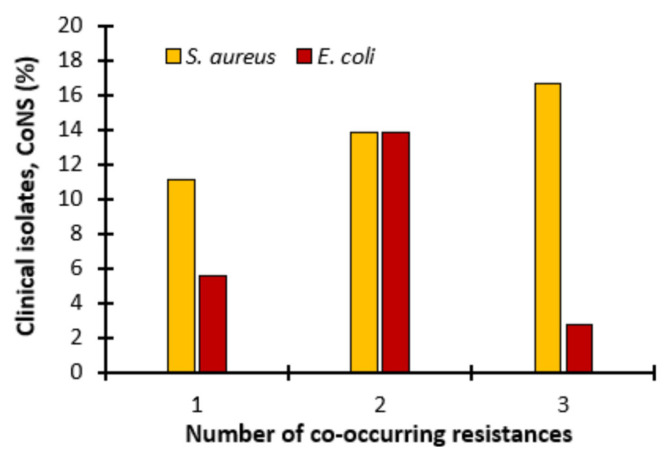
Co-occurring resistance. Transfer of antibiotic resistant determinants to 1, 2 or 3 antibiotics in *S. aureus* 8324−5 and *E. coli* when conjugated with coagulase-negative staphylococci (CoNS).

Additionally, we observed that, in most cases, co-occurring resistance was observed (Figure 5). In the case of transfer to *S. aureus*, as many as 16.67% of donors transferred resistance to three antibiotics, 13.89% to two and 11.11% to one. In turn, in the case of the mobility of resistance determinants to *E. coli*, co-occurring resistance to three antibiotics was rarely observed (2.78%), slightly more often to one (5.56%), and most often to two (13.89%) (Figure 5).

Our findings correspond to previous scientific reports indicating that conjugation within one genus of bacteria is more effective than between unrelated bacteria [14]. However, our results are disturbing because they indicate that the determinants of resistance to antibiotics harbored in CoNS are broad-host-range plasmids and are effectively transferred to *E. coli*, which is a representative of Gram-negative bacteria. These findings are extremely valuable because the transfer of antibiotic resistance determinants between Gram-positive and Gram-negative bacteria is limited in the literature. We found only a few scientific reports in this field. The study by Bes et al. [44] also demonstrated the mobility of resistance determinants between Gram-positive and Gram-negative bacteria. The authors indicated the transfer of plasmid p_8N_qac(MN687830.1) as a potential mechanism for the spread of chlorhexidine resistance between *S. aureus* and *E. coli* [44]. In another study, Manohar et al. [43] assessed the transfer of antibiotic resistance genes between *S. sciuri* and *E. coli* and indicated the spread of antibiotic resistance due to the effective transfer of plasmids carrying antibiotic resistance genes. Thus, our research sheds new light on the problem of antibiotic resistance—elements harboring antibiotic resistance genes can be effectively transferred from Gram-positive bacteria—apparently benign CoNS naturally occurring on human skin and mucous membranes—to other, also unrelated Gram-negative bacteria, i.e., *E. coli*. This effective transfer of antibiotic resistance determinants from CoNS to Gram-negative bacteria indicates the need to take action to control CoNS, especially in hospitals. Gram-negative bacteria are currently a group of pathogens for which there is an urgent lack of effective therapies, and the potential transfer of resistance genes from Gram-positive donors may further enhance this effect, turning them into superbugs.

### 2.5. Susceptibility of CoNS to Antiseptics

Considering the high antibiotic resistance and effective transfer of resistance determinants between CoNS and *S. aureus* and *E. coli*, we decided to check whether the method of skin disinfection before the blood collection procedure (the type of preparation and exposure time) can affect the number of reported BCC cases and the size of the CoNS population carrying antibiotic resistance genes in hospitals and medical settings. Antiseptics routinely used to disinfect skin before blood collection and disinfectants used on skin (Rivanol and Octenidyne) were tested against planktonic cells and biofilms of CoNS. It was shown that disinfectants such as Kodan, Betadine, Rivanol, CITROclorex and Octenidyne completely combated planktonic cells from the UCC and CED groups after just 10 s of exposure, and this effect was also observed after 60 s (Table 6). Thus, all disinfectants routinely used for skin disinfection showed strong activity against planktonic cells.

**Figure 6:**
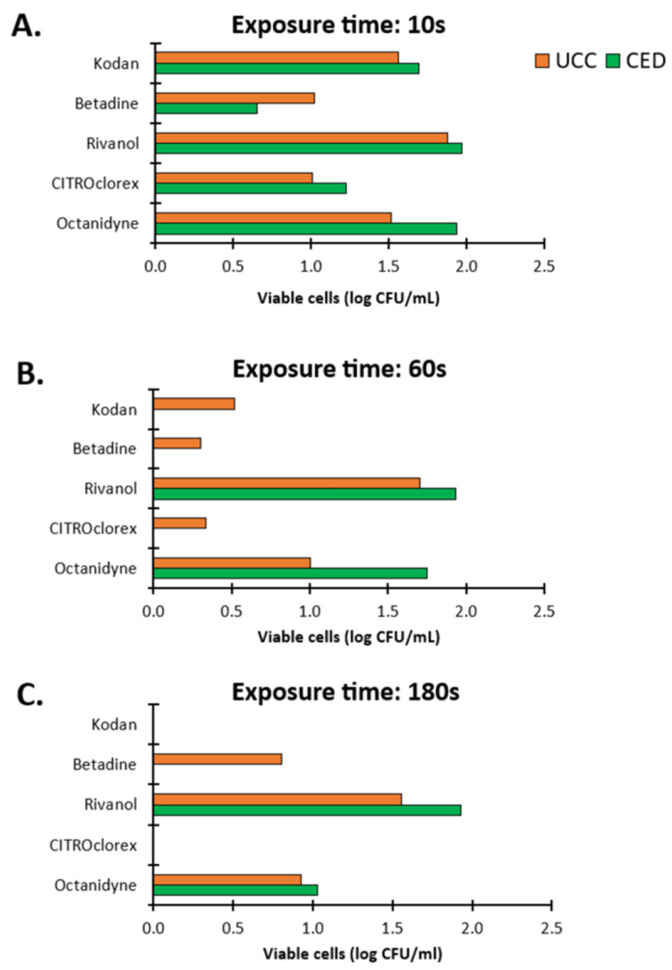
The impact of antiseptics on biofilms of coagulase-negative staphylococci (CoNS) determined as blood culture contamination (BCC) in patients of the University Clinical Center (UCC) and the Clinical Emergency Department (CED) in Gdansk. The results were obtained with a plate count assay (PCA) after exposure times of 10 (**A**), 60 (**B**) and 180 (**C**) s.

The observation was definitely different in the case of biofilms (Figure 6). A ten-second exposure of disinfectants to biofilms resulted in the presence of live cells in the range of 0.65 log CFU/mL to 1.97 log CFU/mL (Figure 6A). The strongest effect within 10 s was demonstrated by Betadine for CoNS from the CED group with live cells of 0.65 log CFU/mL and CITROclorex for CoNS from the UCC group with a 1.01 log CFU/mL of bacterial load. The lowest effect was demonstrated for Rivanol with live cells of 1.88 and 1.97 log CFU/mL for the UCC and CED group, respectively, and for Octenidyne with live cells of 1.51 and 1.94 log CFU/mL for the UCC and CED group, respectively (Figure 6A). After a 60 s treatment of biofilms with Kodan, Betadine and CITROclorex, the complete eradication of biofilm from the CED group was demonstrated and the activity of Rivanol and Octenidyne resulted in live cells of 1.93 and 1.75 log CFU/mL, respectively (Figure 6B). In turn, biofilms of strains from the UCC group showed a growth in the range of 0.30–1.70 log CFU/mL after exposure to Betadine and Rivanol, respectively (Figure 6B). The highest activity was observed for Betadine and CITROclorex with the number of live cells of 0.30 and 0.34 log CFU/mL, respectively. A 180 s exposure to antiseptics showed the strongest antibiofilm effect,; however, under Rivanol and Octenidyne treatment, a bacterial growth of 1.56 and 0.92 log CFU/mL and 1.93 and 1.03 log CFU/mL was still observed for the UCC and CED group, respectively (Figure 6C). Moreover, Betadine was still ineffective against biofilms from the UCC group with a bacterial load of 0.80 log CFU/mL. Thus, extending the exposure time to 180 s allows for the complete eradication of most biofilms, but the effect of Rivanol, Octenidyne and Betadine was still limited. Generally, antiseptics were more effective against biofilms formed by strains from the CED group, where complete eradication was observed after just 60 s of exposure to Kodan, Betadine and CITROclorex, but live cells were still observed after treatment with Rivanol and Octenidyne (1.93 and 1.75 log CFU/mL, respectively) (Figure 6B). Nevertheless, it should be noted that after both 60 and 180 s of exposure to the weakest antiseptics, Rivanol and Octenidyne, the number of live cells was higher for biofilms formed by strains from the CED group (1.93 and 1.03 log CFU/mL) compared to the UCC group (1.56 and 0.92 log CFU/mL) (Figure 6B,C).

Based on the above observations, it can be concluded that the effectiveness of antiseptics depended on the exposure time. The 180 s treatment showed that the most effective against biofilms were Kodan and CITROclorex, and the least effective were Rivanol and Octenidyne. It is worth emphasizing that Rivanol and Octenidyne are not routinely used as antiseptics for skin disinfection prior to blood collection but are nevertheless commonly used as skin disinfectants. Both Kodan and CITROclorex are antiseptics that contain alcohol as an active ingredient. Alcohol preparations are known as good disinfectants, especially when they contain 60–80% alcohol. The mechanism of their action is to disrupt the cell wall and/or damage cell membranes as a result of protein denaturation and lipid dissolution [45]. Kodan contains 2-propanol, 1-propanol and 2-diphenylol and Octenidyne as active substances has dihydrochloride and phenoxyethanol as preservatives. Both propanol and ethanol are short-chain alcohols, and their high antibacterial effect obtained in our study corresponds to previous reports indicating that short-chain alcohols, especially when dissolved in water, are good disinfectants, but their effectiveness depends on the isomer [46]. Moreover, CITROclorex contains chlorhexidyne and this component can be important for antibacterial activity. In the study by Hefzy et al. [47], they also showed the highest activity of antiseptics with chlorhexidyne, but the effect was not that spectacular because the antiseptic with chlorhexidyne showed 80% resistance to CoNS compared to over 90% resistance of the other two tested disinfectants [47]. The study by Ota et al. [48] also showed that antiseptics with alcohol/chlorhexidine are more effective than disinfectants containing povidone iodine, which is reflected in a lower percentage of the contamination of blood cultures [38]. In turn, the study by Garrido-Benedicto et al. [49] showed that in a 24-month prospective study where common soaps (*n* = 108) or body sponges impregnated with 4% chlorhexidine (*n* = 109) were used to clean patients of an intensive care unit, a lower rate of contamination was found in blood samples from patients cleaned with the addition of chlorhexidine [49]. Moreover, a meta-analysis by Caldeira et al. [50] based on the results of randomized controlled trials concluded that alcoholic solutions are generally more effective than non-alcoholic solutions in reducing BCC and that alcoholic chlorhexidine solutions significantly reduce BCC compared to aqueous solutions of povidone iodine and were important in the clinical picture of reducing the number of BCCs in relation to alcoholic iodine [50]. Therefore, our results correspond to previous reports indicating the advantage of alcohol solutions and solutions containing chlorhexidine compared to other antiseptics. Moreover, our research indicates that the selection of a skin disinfectant before blood collection and the duration of exposure to the agent are crucial to reducing false-positive blood cultures. It is reasonable to call for an extension of the exposure time of the disinfectant on the patient’s skin. The use of shorter exposure times does not completely protect against the elimination of bacteria from the skin surface. It leads to false-positive blood culture results and, consequently, to the unnecessary use of antibiotics and extended hospitalization time. In the long term, the futile use of antibiotics causes the development of antibiotic resistance by accustoming microorganisms to living in the environment of antibiotics and leads to the development of mechanisms of resistance to these substances.

**Table 6:** Characteristics of recipients used to assess the mobility of antibiotic resistance determinants from CoNS.

Strain	Description	Source
*E. coli* MG1655	K-12 F- λ- ilvG-rfb-50 rph-1	[51]
*E. coli* MG1655 Tc^R^	*ortT*::Tet^R^	Unpublished ^a^
*E. coli* DH5α Rif^R^	F^−^ φ80lacZΔM15 Δ(lacZYA-argF)U169 recA1 endA1 hsdR17(rK^−^ mK^+^) phoA supE44 thi-1 gyrA96 relA1 Rif^r^	[52]
*E. coli* HB101 Str^R^	F^−^ hsdS20(rB^−^ mB^−^) recA13 ara-14 proA2 lacY1 galK2 rpsL20(StrR) xyl-5 mtl-1 supE44 λ^−^	[53]

^a^ The strain of *E. coli* MG1655 Tc^R^ was used in the study as a courtesy of Dr. Ewa Wons from the Department of Microbiology, the Faculty of Biology, the University of Gdansk.

## 3. Conclusions

Blood culture contamination is a problem often reported in hospitals and medical facilities. Our findings indicate that CoNS, often ignored, have a high virulence potential due to their ability to form biofilms, resistance to antibiotics and high mobility of antibiotic resistance determinants between phylogenetically related and distant bacteria. Moreover, we showed the effective transfer of antibiotic resistance genes between CoNS and Gram-negative *E. coli*, thus indicating a broad range host of antibiotic resistant determinants. Therefore, the results of our research shed new light on previously benign CoNS and indicate the need to control them, especially in hospitals struggling with the problem of antibiotic resistance, because the potential transfer of antibiotic resistance determinants to other bacteria, which we demonstrated in this study, may intensify the phenomenon of antibiotic resistance and impede effective treatment. Our results also indicate that the careful selection of skin disinfectants before blood collection and the prolongation of exposure time can eliminate the number of reported BCCs and reduce the spread of high-virulence CoNS in hospitals and medical facilities.

## 4. Materials and Methods

Consent to conduct this research was obtained from the Scientific Research Committee of the Medical University of Gdansk (KB/246/07.07.2023). Patient blood samples were collected from 1 December 2022 to 31 March 2023. Blood samples were collected from adult patients (>18 years of age) admitted to hospital departments (the University Clinical Center, UCC) and to the Clinical Emergency Department (CED). This study did not analyze or examine patients; therefore, consent for testing was not sought from the study participants (patients). These were not prospective studies and we did not collect samples from patients for research purposes. Blood for culture was routinely collected in accordance with the course of the clinical procedure. During the study, we did not have access to data allowing the identification of patients. Furthermore, we did not retrospectively analyze medical records and archived samples.

### 4.1. Strain Isolation and Identification

All 36 strains used in this study were isolated from 1 December 2022 to 31 March 2023 from patients different units of the University Clinical Center in Gdansk (UCC, 17 isolates) and Clinical Emergency Department in Gdansk (CED, 19 isolates). The factor differentiating the patients was the time spent in a medical facility and in the case of UCC patients it was over 48 h, while in the case of CED patients it was 48 h or less. The BCC criterion was the isolation of a strain or more than one strains from 1 bottle when 2 or more bottles were collected in one time from a patient without implanted artificial devices, prostheses or catheters. The blood samples were collected during the routine procedure by qualified medical personnel. Strains used in this study were the effect of false-positive blood culture referred to as blood culture contaminants (BCC). These strains were identified using MALDI-TOF MS (MALDI biotyper; Bruker Daltonics, MA USA) according to the manufacturer’s instructions.

### 4.2. The Ability to Form Biofilms

The ability to form biofilms by BCC was determined using crystal violet (CV) staining according to Al-Bayati and Samarasinghe [18]. Briefly, an overnight culture of bacteria was diluted 10 times and grown in fresh nutrient broth (NB) to OD_600_ = 0.1. Then, 200 µL of bacterial suspension was placed in a 96-well microtiter plate (Nunclon Delta Surface, Thermo Scientific, Waltham, MA USA) and incubated at 37 °C. After 24 h, the medium with planktonic, non-adherent cells was removed and the wells were rinsed twice with 200 µL of sterile phosphate-buffered saline (PBS). The biofilm formed in wells was stained with 200 µL of 1% (*w*/*v*) CV (Sigma Aldrich, Hamburg, Germany) for 20 min at room temperature. Then, the wells were rinsed with 200 µL of PBS, and next, the dye was diluted with 200 µL of 33% (*v*/*v*) acetic acid (Sigma Aldrich, Germany). Finally, the OD was measured at 595 nm (EPOCH, BioTek, Warsaw, Poland) to evaluate the load of the formed biofilm. The medium (NB) was used as a negative control and this result was used to calculate the final results. The strains were classified according to Al-Bayati and Samarasinghe [12] as strong biofilm producers (OD_595_ > 0.5), moderate biofilm producers (OD_595_ 0.3–0.5), weak biofilm producers (OD_595_ < 0.3), a lack of biofilm formation (OD_595_ < 0.15) and non-adherent strains (OD_595_ = 0).

### 4.3. Determination of Antibiotic Resistance

The antibiotic resistance of the tested strains was evaluated using a disk diffusion method recommended by Clinical Laboratory Standards Institute (CLSI) guidelines. Antibiotics were purchased from OXOID (GB) and used at clinical concentrations as follows: cefoxitin (FOX): 30 µg/mL; penicillin (PG): 1 µg/mL; clindamycin (CC): 2 µg/mL; erythromycin (E): 15 µg/mL; co-trimaxazole (SXT): 1.25–23.75 µg/mL; ciprofloxacin (CIP): 5 µg/mL; vancomycin (VA): 5 µg/mL; rifampicin (RIF): 5 µg/mL; and linezolid (LNX): 10 µg/mL. Plates were incubated overnight (16–18 h, against VA, full 24 h) at 37 °C and then the zone sizes were measured. Going further, we assessed the antibiotic resistance of the tested strains to antibiotics at standardized concentrations typically employed in microbiological assays as follows: ampicillin (AMP): 100 µg/mL; kanamycin (KAN): 50 µg/mL; chloramphenicol (CM): 30 µg/mL; rifampicin (RIF): 50 µg/mL; ciprofloxacin (CIP): 5 µg/mL; gentamicin (GEN): 40 µg/mL; streptomycin (STR) 25 µg/mL; and tetracycline (TET): 15 µg/mL. Growth media, Luria broth (LB) or Luria agar (LA), were supplemented with appropriate antibiotics, and the strains were placed onto plates. After 24 h of incubation (37 °C), based on bacterial growth or growth inhibition, an antibiogram for each strain was performed.

### 4.4. Mobilization Potential of Antibiotic Resistance Determinants

The high percentage of antibiotic-resistant strains encouraged us to investigate the mobility of antibiotic resistance determinants between CoNS (donors) and *S. aureus* and *E. coli* (recipients), thereby determining gene transfer between a related Gram-positive strain (*S. aureus*) and a representative of Gram-negative bacteria (*E. coli*). The strains used as recipients are characterized in Table 6. Before beginning a conjugation assay, each donor strain was plated on MSA (mannitol salt agar *aka* Chapman medium) and MacConkey agar to confirm that the donors growing on MSA do not grow on MacConkey agar, on which grow *E. coli* (recipient) and vice versa. *S. aureus* (StrR—streptomycin-resistant; recipient) also grow on MSA but colonies can be distinguished because *S. aureus* can metabolize mannitol, and due to acidification, *S. aureus* discolors the growth medium, and most of donor strains do not metabolize mannitol, and the growth medium remains unchanged. As the recipient was resistant to streptomycin, for conjugation, we selected strains sensitive to this antibiotic. For the second recipient, *E. coli*, three strains were tested, DH5α RifR, HB101 StrR, and MG1655 TcR, resistant to rifampicin (RIF), streptomycin (STR), and tetracycline (TET), respectively. The antibiotic resistance profiles of these strains were confirmed to select probable mobile resistances for testing. A conjugation assay was performed according to Bes et al. (2021) [44] with modifications. Briefly, for conjugation matings, donors and recipients were grown overnight in brain heart infusion (BHI) broth supplemented with antibiotics specific to the resistance they encoded. Then, the culture was diluted 50-fold in fresh BHI and grown to an OD_600_ of 0.5. The donor and recipient cultures were concentrated 10-fold by centrifugation and resuspended in fresh BHI. The matings were performed on solid media by mixing equal volumes of donor and recipient strains and transferring the mixture to a conjugation filter (0.22 µm nitrocellulose, Millipore) on a BHI agar plate. The mating mixtures were then incubated at 37 °C for 18 h. Afterward, the filter was resuspended in PBS, and appropriate dilutions were plated on selective media for donors, recipients and transconjugants. In the first experiment, when we used *S. aureus* as the recipient, the recipient strains were plated on MSA plates to count the recipient population. In this case, transconjugants were plated on MSA with antibiotics for the recipient (STR) and the appropriate antibiotics corresponding to the resistance encoded by the donor. Considering, that the possible mobile genetic element was unknown, we used each resistance carried by the donor separately. In the case where *E. coli* was used as a recipient, the recipients were plated on MacConkey agar with each antibiotic resistance possessed by the donor, as mentioned above. The frequency of conjugation is expressed as the number of transconjugants per recipient cell.

### 4.5. Activity of Antiseptics on Planktonic Cells

The activity of antiseptics used to prepare skin before blood collection or disinfectants used on skin (Rivanol and Octenidyne) was performed using the filtration method in accordance with the European Standard (EN 13727:2012+A2) [54]. Tests conducted using the method/version utilized ready-to-use preparations. Briefly, the overnight culture of bacteria was added to 5 mL of NB to obtain a density of 10 in the McFarland scale. Then, 300 µL of bacterial suspension was added to 9700 µL of tested disinfectant: Kodan (SCHULKE & MAYR GmbH, Norderstedt, Germany), 10% Betadine (EGIS, Warsaw, Poland), 0.1% Rivanol (DERMAPHARM AG, Grünwald, Germany), 2% CITROclorex (ECOLAB, Cracow, Poland) and Octenidyne (SCHULKE & MAYR GmbH, Norderstedt, Germany). The exposure time was 10 s, 1 min and 3 min. After this time, 100 µL of neutralizer was added [54]. The neutralizers were as follows: for alcohol-based antiseptics,–polysorbate80 30 g/L + saponin 30 g/L + lecithin 3 g/L/and rinsing liquid–tryptone 1 g/L + NaCl 9 g/L + polysorbate80 5 g/L; for iodine-based antiseptics,–sodium thiosulfate 3 g/L + polysorbate80 30 g/L + lecithin 3 g/L and rinsing liquid–sodium thiosulfate 3 g/L (EN 13727:2012+A2). Then, the whole volume was filtered through a membrane filter with a pore diameter of 0.45 µm. The filters were transferred to nutrient agar (NA) plates (Graso Biotech, Starogard Gdanski, Poland) and incubated for 24 h at 37 °C. Finally, the grown colonies were counted and the results were expressed as log CFU/mL.

### 4.6. Activity of Antiseptics on Biofilms

The activity of disinfectants on biofilms was determined against biofilms formed in 24-well microtiter plates (Nunclon Delta Surface, Thermo Scientific). For this purpose, 100 µL bacterial culture (OD_600_ 0.5) was placed in each well with 900 µL of fresh NB. The content of the wells were vigorously mixed and the plate was incubated under static conditions for 72 h at 37 °C. After this, the medium with non-adhered cells was removed and the biofilm was washed gently with 1 mL of PBS. Then, the biofilm was covered with 1 mL of the tested disinfectant: Kodan, Betadine, Rivanol, CITROclorex and Octenidyne for 10 s, 1 min and 3 min. After the given exposure time, the disinfectant was removed and 1 mL of neutralizer was added in accordance with EN 13727:2012+A2 and described above. After 3 min, the neutralizer was removed and the biofilm was rinsed with 1 mL of PBS. Then, the biofilm was harvested using a sterile swab, suspended in 1 mL of sterile saline (0.85% NaCl) and vigorously vortexed for 1 min. A series of ten-fold dilutions were made and 100 µL of appropriate dilutions were spread onto the NA plates. The plates were incubated for 24 h at 37 °C. Finally, the grown colonies were counted and the results were expressed as log CFU/mL.

## Figures and Tables

**Table 1:** Strains used in the study. Clinical isolates obtained as blood culture contamination (BCC) from patients of the University Clinical Center (UCC) and the Clinical Emergency Department (CED) in Gdansk.

Number	ID	CoNS
UCC group		
1	3404	*S. epidermidis*
2	3405	*S. epidermidis*
3	3406	*S. epidermidis*
4	3812	*S. epidermidis*
5	3433	*S. haemolyticus*
6	3819	*S. epidermidis*
7	3346	*S. saprophiticus*
8	3340	*S. epidermidis*
9	3409	*S. epidermidis*
10	3450	*S. epidermidis*
11	3434	*S. haemolyticus*
12	3448	*S. epidermidis*
13	3821	*S. hominis*
14	3437	*S. epidermidis*
15	3436	*S. epidermidis*
16	3342	*S. epidermidis*
17	3407	*S. epidermidis*
CED group		
18	3212	*S. epidermidis*
19	3171	*S. hominis*
20	3173	*S. epidermidis*
21	3184	*S. epidermidis*
22	3187	*S. epidermidis*
23	3217	*S. epidermidis*
24	3209	*S. epidermidis*
25	3210	*S. epidermidis*
26	3188	*S. epidermidis*
27	3211	*S. epidermidis*
28	3213	*S. epidermidis*
29	3215	*S. epidermidis*
30	3170	*S. epidermidis*
31	3216	*S. capitis*
32	3214	*S. epidermidis*
33	3189	*S. capitis*
34	2121261/2	*S. simulans*
35	3702	*S. epidermidis*
36	3449	*S. epidermidis*

**Table 2:** The distribution of individual species of coagulase-negative staphylococci (CoNS) among blood culture contamination (BCC) samples. The division was made by dividing patients of the University Clinical Center (UCC) and the Clinical Emergency Department (CED) in Gdansk.

	Source	Frequency	Percentage (%)
*S. epidermidis*	UCC	13	76.47
	CED	15	78.95
	**Total**	28	**77.78**
*S. haemolyticus*	UCC	2	11.76
	CED	0	0
	**Total**	2	**5.56**
*S. saprophiticus*	UCC	1	5.88
	CED	0	0
	**Total**	1	**2.78**
*S. hominis*	UCC	1	5.88
	CED	1	5.26
	**Total**	2	**5.56**
*S. capitis*	UCC	0	0
	CED	2	10.52
	**Total**	2	**5.56**
*S. simulans*	UCC	0	0
	CED	1	5.26
	**Total**	1	**2.78**

**Table 3:** The antibiotic profile of CoNS from the UCC and CED, where R stands for resistant and S stands for sensitive (left), and the mobility of resistance genes between clinical isolates of CoNS and *S. aureus* 8324-5 (right). Streptomycin-resistant strains are marked in orange and are excluded from further tests due to the recipient’s resistance (*S. aureus* 8324-5) to this antibiotic. Strains marked in blue are qualified for conjugation. The transfer of resistance genes is marked in green, different green intensities indicate the presence of resistance genes on other mobile genetic elements, and the lack of mobility of resistance genes is defined as “no” and marked in yellow. The antibiotics used are as follows: ampicillin (AMP), kanamycin (KAN), chloramphenicol (CM), and ciprofloxacin (CIP).

	Antibiotic Resistance						Strain with Identifier		Checking Mobility Resistance—Recipient: *S.aureus* 8324-5				
	AMP	KAN	CM	CIP	GEN	STR	ID	Strain	AMP	KAN	CM	CIP	GEN
**UCC**	S	S	S	S	S	S	3404	*S. epidermidis*					
	S	R	S	R	R	S	3405	*S. epidermidis*		1.3 ± 0.9 × 10^−2^		no	1.3 ± 1.2 × 10^−3^
	S	R	R	R	R	S	3406	*S. epidermidis*		2.2 ± 1.8 × 10^−6^	no	no	no
	R	R	S	R	R	S	3812	*S. epidermidis*	no	5.7 ± 4.0 × 10^−3^		3.7 ± 1.4 × 10^−4^	4.8 ± 1.0 × 10^−3^
	R	R	S	R	R	R	3433	*S. haemolyticus*					
	S	R	S	R	R	S	3819	*S. epidermidis*		9.5 ± 4.8 × 10^−3^		1.1 ± 1.0 × 10^−4^	1.8 ± 0.6 × 10^−3^
	S	S	S	S	S	S	3346	*S. saprophyticus*					
	S	S	S	S	S	S	3340	*S. epidermidis*					
	R	S	R	S	S	R	3409	*S. epidermidis*					
	S	R	R	S	R	S	3450	*S. epidermidis*		5.5 ± 4.9 × 10^−4^	9.7 ± 6.3 × 10^−4^		no
	R	R	R	R	R	R	3434	*S. haemolyticus*					
	S	R	R	S	R	S	3448	*S. epidermidis*		no	no		no
	S	R	R	R	R	S	3821	*S. hominis*		7.9 ± 4.2 × 10^−3^	2.0 ± 3.1 × 10^−3^	5.7 ± 3.2 × 10^−3^	no
	S	R	S	R	R	S	3437	*S. epidermidis*		3.5 ± 2.7 × 10^−1^		2.7 ± 2.5 × 10^−4^	1.6 ± 1.3 × 10^−1^
	R	R	R	S	R	R	3436	*S. epidermidis*					
	S	S	S	S	S	S	3342	*S. epidermidis*					
	R	R	R	S	S	R	3407	*S. epidermidis*					
**CED**	S	R	R	S	S	S	3212	*S. epidermidis*		4.6 ± 3.8 × 10^−2^	1.7 ± 1.5 × 10^−4^		
	S	S	S	S	S	S	3171	*S. hominis*					
	S	S	S	S	S	S	3173	*S. epidermidis*					
	S	S	S	S	S	S	3184	*S. epidermidis*					
	R	R	R	S	S	R	3187	*S. epidermidis*					
	S	S	S	S	S	S	3217	*S. epidermidis*					
	S	R	S	R	R	S	3209	*S. epidermidis*		3.6 ± 2.4 × 10^−4^		no	no
	R	R	S	S	S	R	3210	*S. epidermidis*					
	R	R	S	S	R	R	3188	*S. epidermidis*					
	S	S	S	R	S	S	3211	*S. epidermidis*				1.3 ± 0.5 × 10^−5^	
	S	S	R	S	S	S	3213	*S. epidermidis*			5.9 ± 4.2 × 10^−4^		
	S	R	S	S	S	S	3215	*S. epidermidis*		5.4 ± 2.9 × 10^−4^			
	S	S	S	S	S	S	3170	*S. epidermidis*					
	R	R	S	S	R	S	3216	*S. capitis*	2.1 ± 2.0 × 10^−2^	1.6 ± 1.4 × 10^−2^			6.7 ± 2.8 × 10^−2^
	R	S	S	R	S	S	3214	*S. epidermidis*	1.6 ± 1.4 × 10^−3^			4.6 ± 3.0 × 10^−4^	
	S	S	S	S	S	S	3189	*S. capitis*					
	S	R	S	S	S	S	2121261	*S. simulans*		3.6 ± 2.7 × 10^−3^			
	R	R	R	S	S	S	3702	*S. epidermidis*	3.9 ± 2.4 × 10^−2^	3.2 ± 2.0 × 10^−3^	8.0 ± 3.5 × 10^−3^		
	S	S	S	S	S	S	3449	*S. epidermidis*					

**Table 4:** The antibiotic profile of CoNS from the UCC and CED, where R stands for resistant and S stands for sensitive (left), and the mobility of resistance genes between clinical isolates of CoNS and *E. coli* (right). The choice of recipient *E. coli* (*E. coli* MG1655, *E. coli* MG1655 TcR, *E. coli* DH5α RifR, or *E. coli* HB101 StrR) was determined by the resistance profile of the donors. The strains marked in blue were qualified for conjugation, the rest did not show resistance to the antibiotics used. The transfer of resistance genes is marked in green and the lack of the mobility of resistance genes is defined as “no” and marked in yellow. The antibiotics used were as follows: ampicillin (AMP), kanamycin (KAN), chloramphenicol (CM), rifampicin (RIF), ciprofloxacin (CIP), gentamycin (GEN), streptomycin (STR), and tetracycline (TET).

	Antibiotic Resistance								Strain with Identifier		Checking Mobility Resistance—Recipnemt: *E. coli*						
	AMP	KAN	CM	RIF	CIP	GEN	STR	TET	ID	Strain	AMP	KAN	CM	CIP	GEN	STR	TET
**UCC**	S	S	S	R	S	S	S	S	3404	*S. epidermidis*							
	S	R	S	S	R	R	S	S	3405	*S. epidermidis*		no		no	no		
	S	R	R	R	R	R	S	S	3406	*S. epidermidis*		no	no	no	no		
	R	R	S	R	R	R	S	S	3812	*S. epidermidis*	no	no		no	no		
	R	R	S	R	R	R	R	S	3433	*S. haemolyticus*	no	1.7 ± 1.2 × 10^−4^		no	no	7.4 ± 5.49 × 10^−5^	
	S	R	S	S	R	R	S	S	3819	*S. epidermidis*		no		no	no		
	S	S	S	S	S	S	S	S	3346	*S. saprophyticus*							
	S	S	S	S	S	S	S	S	3340	*S. epidermidis*							
	R	S	R	S	S	S	R	S	3409	*S. epidermidis*	no		no			no	
	S	R	R	S	S	R	S	S	3450	*S. epidermidis*		4.3 ± 3.2 × 10^−3^	6.4 ± 1.9 × 10^−3^		no		
	R	R	R	S	R	R	R	R	3434	*S. haemolyticus*	no	6.0 ± 3.9 × 10^−4^	no	no	no	no	no
	S	R	R	R	S	R	S	S	3448	*S. epidermidis*		no	no		no		
	S	R	R	S	R	R	S	S	3821	*S. hominis*		no	no	no	no		
	S	R	S	S	R	R	S	S	3437	*S. epidermidis*		8.93 ± 0.55 × 10^−3^		no	2.3 ± 0.7 × 10^−3^		
	R	R	R	R	S	R	R	S	3436	*S. epidermidis*	no	no	no		no	no	
	S	S	S	S	S	S	S	S	3342	*S. epidermidis*							
	R	R	R	S	S	S	R	S	3407	*S. epidermidis*	no	no	no			no	
**CED**	S	R	R	S	S	S	S	S	3212	*S. epidermidis*		no	no				
	S	S	S	S	S	S	S	S	3171	*S. hominis*							
	S	S	S	S	S	S	S	S	3173	*S. epidermidis*							
	S	S	S	S	S	S	S	S	3184	*S. epidermidis*							
	R	R	R	S	S	S	R	S	3187	*S. epidermidis*	5.4 ± 1.9 × 10^−4^	no	2.9 ± 2.4 × 10^−4^			no	
	S	S	S	S	S	S	S	S	3217	*S. epidermidis*							
	S	R	S	R	R	R	S	S	3209	*S. epidermidis*		no		no	no		
	R	R	S	S	S	S	R	S	3210	*S. epidermidis*							
	R	R	S	S	S	R	R	S	3188	*S. epidermidis*	no	no			no	no	
	S	S	S	S	R	S	S	S	3211	*S. epidermidis*				no			
	S	S	R	S	S	S	S	S	3213	*S. epidermidis*			no				
	S	R	S	S	S	S	S	S	3215	*S. epidermidis*		no					
	S	S	S	S	S	S	S	S	3170	*S. epidermidis*							
	R	R	S	S	S	R	S	S	3216	*S. capitis*	2.7 ± 2.2 × 10^−3^	1.2 ± 1.1 × 10^−3^			1.8 ± 1.2 × 10^−3^		
	R	S	S	S	R	S	S	S	3214	*S. epidermidis*	no			no			
	S	S	S	S	S	S	S	S	3189	*S. capitis*							
	S	R	S	S	S	S	S	S	2121261	*S. simulans*		1.8 ± 0.9 × 10^−3^					
	R	R	R	S	S	S	S	S	3702	*S. epidermidis*	2.3 ± 1.6 × 10^−4^	1.3 ± 1.0 × 10^−4^	no				
	S	S	S	S	S	S	S	S	3449	*S. epidermidis*							

**Table 5:** Results of antiseptic treatment. Effect of antiseptics on planktonic cells of coagulase-negative staphylococci (CoNS) determined as blood culture contamination (BCC) in patients of University Clinical Center (UCC) and Clinical Emergency Department (CED) in Gdansk.

Antiseptic	Time (s)	Group	Live Cells (log CFU/mL)
Kodan	10	UCC	0
		CED	0
	60	UCC	0
		CED	0
Betadine 10%	10	UCC	0
		CED	0
	60	UCC	0
		CED	0
Rivanol 0.1%	10	UCC	0
		CED	0
	60	UCC	0
		CED	0
CITROclorex 2%	10	UCC	0
		CED	0
	60	UCC	0
		CED	0
Octenidyne	10	UCC	0
		CED	0
	60	UCC	0
		CED	0

## Data Availability

Data will be made available on request and the original data are deposited in repOD at the following link: https://doi.org/10.18150/O72VL6 (18 March 2025).
